# The association between non-alcoholic fatty liver disease (NAFLD) and advanced fibrosis with blood selenium level based on the NHANES 2017-2018

**DOI:** 10.1080/07853890.2022.2110277

**Published:** 2022-08-17

**Authors:** Jie Liu, Liao Tan, Zhaoya Liu, Ruizheng Shi

**Affiliations:** a Department of Cardiovascular Medicine, Xiangya Hospital, Central South University, Changsha, China; b Department of Cardiology, The Third Xiangya Hospital, Central South University, Changsha, China; c Department of the Geriatrics, The Third Xiangya Hospital, Central South University, Changsha, China

**Keywords:** Non-alcoholic fatty liver disease, NAFLD, blood selenium level, National Health and Nutrition Examination Survey, NHANES, advanced liver fibrosis

## Abstract

**Background & Objective:**

Selenium was one of the essential trace elements that played a pivotal role in human health. Although previous studies have investigated the relationship between selenium and non-alcoholic fatty liver disease (NAFLD) and fibrosis, these findings were still inconclusive. Our study was aimed to explore the association between blood selenium level and NAFLD and advanced liver fibrosis diagnosed by vibration controlled transient elastography (VCTE) in US adults.

**Methods:**

All data were extracted from National Health and Nutrition Examination Survey database (2017–2018). Participants were divided into four groups according to quartile of blood selenium level. Liver stiffness and controlled attenuation parameter (CAP) were measured by VCTE. Multiple logistic regression models and subgroup analyses were conducted to determine the association between blood selenium level and NAFLD and advanced liver fibrosis diagnosed by a variety of methods.

**Results:**

A total of 3336 participants were enrolled in main analysis. In multiple logistic regression models, the higher blood selenium level (>205.32, ≤453.62 μg/L) had a significant positive association with NAFLD (β = 1.31). Moreover, high blood selenium level had significantly inversely association to advanced liver fibrosis (β = 0.61). In subgroup analysis, the main inversely correlation between blood selenium and advanced liver fibrosis was found in males with high blood selenium level. Despite dietary selenium intake being adjusted or in different subgroups, the associations between blood selenium level and NAFLD/advanced liver fibrosis remained significant.

**Conclusions:**

This study showed that blood selenium level were positively association with NAFLD among US population. Participants with lower blood selenium level showed a higher percentage of advanced liver fibrosis. Blood selenium is more likely to cause NAFLD and liver fibrosis due to imbalances in selenium homeostasis rather than dietary selenium intake.

**Key messages**
- High blood selenium level was association with NAFLD diagnosed by vibration controlled transient elastography.
- Participants with lower blood selenium level had high percentage of advanced liver fibrosis.
- NAFLD and liver fibrosis are caused by an imbalance of selenium homeostasis, not by dietary selenium intake.

## Introduction

There are over 80 million people diagnosed with non-alcoholic fatty liver disease (NAFLD) in America [1]. The incidence of NAFLD has steadily increased in recent years as a result of changes of dietary, lifestyle, and health, which leaded to severe socioeconomic burdens [2]. NAFLD was a progressive disease spectrum including simple liver steatosis, non-alcoholic steatohepatitis (NASH), cirrhosis, and hepatocellular carcinoma (HCC) [3]. Multiple pathophysiological processes contributed to the development of NAFLD, including lipotoxicity, inflammation, oxidative stress, dysbiosis, and gut-liver axis [4].

Approximately 38% NASH patients would develop to liver fibrosis, even cirrhosis and HCC [5]. In NAFLD, liver fibrosis was activated sustainably by long-term chronic hepatic parenchymal injury, inflammation, oxidative stress and lipotoxicity [6]. Therefore, liver fibrosis was considered as the main predictor of mortality and complications of liver diseases [7].

Selenium was one of the essential trace elements that played a pivotal role in human health [8]. The main source of selenium was dietary intake [9]. Absorption from intestinal lumen, dietary selenium incorporated into amino acid. In human proteins, selenium was usually presented as selenocysteine (SeCys) to perform its biological function [10]. According to the gene sequence research, the human selenoproteome contains 25 selenoproteins, including selenoprotein P (SeIP), glutathione peroxidases (GPxs) family, thioredoxin reductases (TrxRs) family, etc [11]. SeIP, generated by liver cells, converted selenium from liver to other organs [12]. Other selenoproteins displayed antioxidative activity that modulates reactive oxygen species (ROS) generation, apoptosis, endoplasmic reticulum (ER) stress and various pathophysiological processes [13]. However, recent research suggested that although low selenium level caused oxidative stress and metabolic disorders in various diseases, high selenium level also enhanced the prevalence of hypertension, dyslipidaemia, diabetes etc [14]. Therefore, more studies are demanded to further confirm the role of selenium in human diseases.

Some animal experiments and clinical researches have explored the role of selenium in NAFLD and liver fibrosis but presented different conclusions. Several studies showed diets with selenium supplement could increase the activity of selenoproteins in the liver and improved liver steatosis, injury and fibrosis in NAFLD mice models [15]. In contrast, epidemiological studies have shown that high blood selenium or dietary selenium intake was associated with an increased prevalence of NAFLD [16,17]. Meanwhile, most researches defined NAFLD by serum activity of Alanine Aminotransferase/Aspartate Aminotransferase (ALT and AST) and Fatty liver index (FLI) merely. But serum activity of ALT & AST was not a reliable indicator for diagnosing NAFLD as up to 80% of NAFLD patients had normal level, and several advanced NAFLD patients had low level [18–20]. FLI was an algorithm included body mass index (BMI), waist circumference, triglycerides and gamma-glutamyl-transferase (GGT) [21]. However, FLI was more likely a useful index to predict the prognosis of metabolic syndrome and had low sensitivity to diagnose NAFLD [22]. Simultaneously, with increasing of blood selenium level, the all-cause mortality decreased in NAFLD patients with liver fibrosis diagnosed by the NAFLD fibrosis score (NFS) which based on age, blood sugar, BMI, platelets, albumin, AST/ALT [23]. Other commonly used diagnostic parameters for advanced liver cirrhosis in clinical studies include Fibrosis-4 index (FIB-4) and Body mass index, AST/ALT Ratio, and Diabetes score (BARD) [24]. FIB-4 also based on age, AST, ALT, platelets [25]. Nonetheless, NFS/BARD/FIB-4, which were indirect diagnostic methods related to blood biomarker of lipid metabolism, had lower effective to diagnose advanced fibrosis compared to vibration controlled transient elastography (VCTE) (Area Under the Receiver Operating Characteristic curve (AUROC): 0.751/0.698/0.787 vs 0.849) [26,27]. Thus, more accurate diagnose methods and more population researches contribute to clear the association between the blood selenium level and NAFLD.

Liver stiffness (LS) and controlled attenuation parameter (CAP) were accurate parameters to reflect liver fibrosis (sensitivity: 93.7%, specificity: 91.1%) and liver steatosis (AUROC: 0.96) detected by VCTE, which a non-invasive diagnosed method [28,29]. National Health and Nutrition Examination Survey (NHANES) was a large clinical program of health and nutrition in U.S. In 2017–2018 cycle, 4266 participants underwent VCTE in NHAENS. Therefore, we utilized VCTE (direct method) to explore the association between blood selenium level and NAFLD and liver fibrosis diagnosed using NHANES 2017–2018 participants, combined four indirect but classic method to further estimate this independent relationship.

## Materials and methods

### Study population

**Figure 1.**
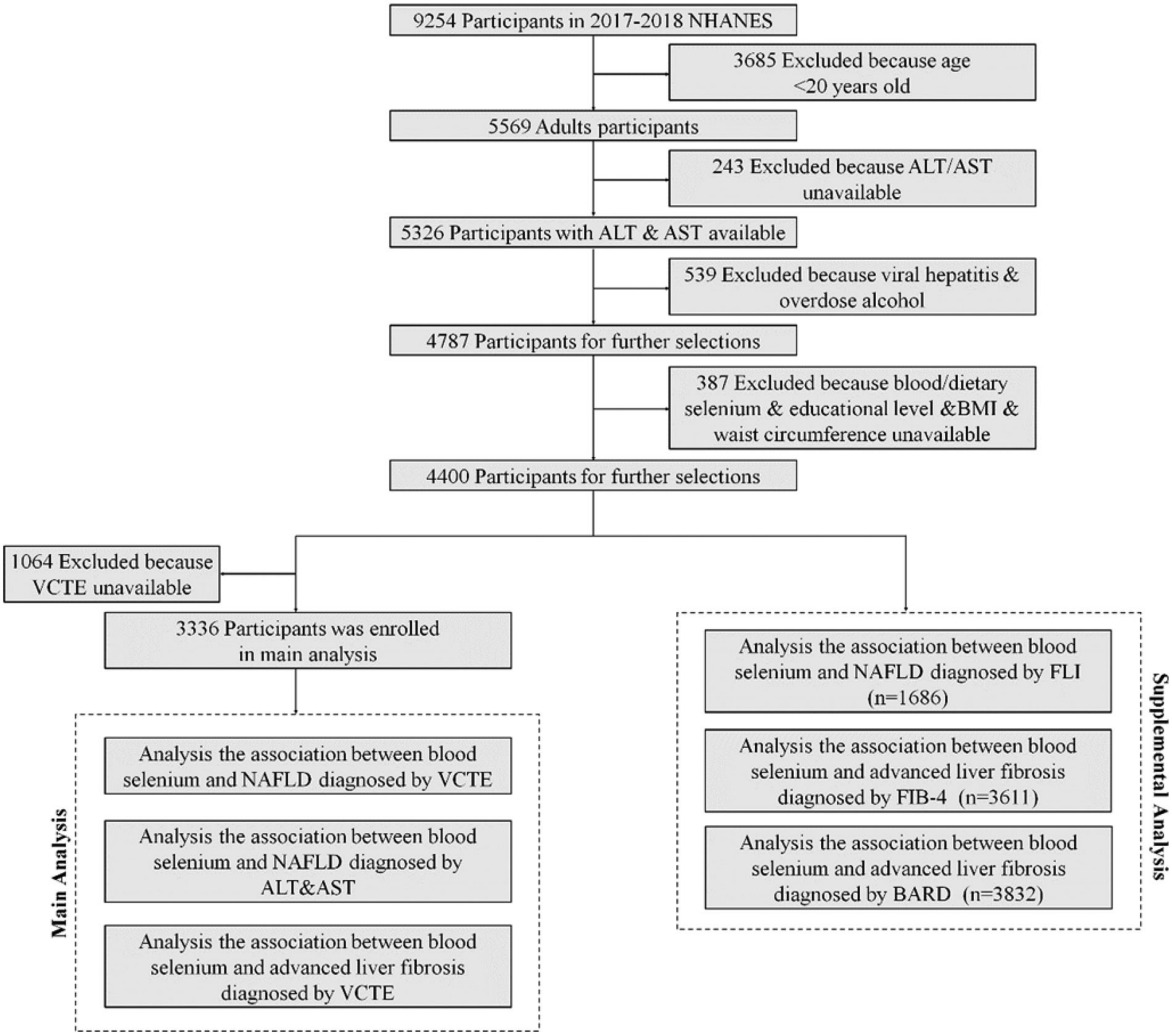
Study flowchart.

The data was obtained from the NHANES database (2017–2018). NHANES, a multistage and complex study, includes demographic, socioeconomic, dietary, health-related questionnaires, and examination data, conducted by National Centre for Health Statistics (NCHS). Among 9254 participants in NHANES 2017-2018, there are 5569 adult participants (>20 years) enrolled. Thereinto, 5326 participants finished measurement of the level of ALT & AST. A total 4787 participants remained after exclusion 539 participants with viral hepatitis and excessive alcohol consumption. Viral hepatitis was defined by positive hepatitis B virus surface antigen (HBsAg) and hepatitis C virus (HCV) RNA. Participants who consumed ≥30 g/d alcohol (male) or ≥20 g/d alcohol (female) were defined as excessive alcohol consumption^[^^30^^]^. Participants with missing data of blood/dietary selenium, education level, body mass index (BMI), waist circumference were further excluded. 4400 participants remained for further selection. After excluded 1064 participants with unavailable data of VCTE, 3336 participants were enrolled in this study and were divided to four groups according to the quartile of concentration of blood selenium for main analysis. In supplementary analysis, 1686 participants were enrolled in exploring the association between blood selenium level and NAFLD diagnosis by FLI. The analysis of the association blood selenium and advanced liver fibrosis diagnosed by FIB-4 and BARD enrolled 3611 and 3832 participants (Figure 1). NHANES 2017-2018 was approval by The NCHS Ethics Review Board, and inform consent was obtained from all participants. The acquisition and analysis of data was consistent with NHANES research requirements.

### NAFLD and advanced liver fibrosis assessments

Liver stiffness and CAP (a novel physical parameter related to liver steatosis) were detected by VCTE using FibroScan model 502 V2 Touch (Echosens, North America) with a medium (M) or extra-large (XL) wand (probe) in the NHANES Mobile Examination Centre (MEC). The activity of AST and ALT were measured using the kinetic rate method on the Roche Cobas 6000 (c501 module) analyser. FLI was ranged from 0 to 100 using the followed formula: (e ^0.953^*^loge (triglycerides) + 0.139*BMI + 0.718*loge (ggt) + 0.053*waist circumference − 15.745^)/(1 + e ^0.953*loge (triglycerides) + 0.139*BMI + 0.718*loge (ggt) + 0.053*waist circumference − 15.745^) * 100. FIB-4 was calculated by followed formula: age (years) × AST [U/L]/(platelets [10^9^/L] × (ALT [U/L])1/2). BARD was ranged from 0 to 4 defined by BMI, AST/ALT ratio and history of diabetes. BMI ≥28 kg/m^2^ was given 1 point. AST/ALT ratio ≥0.8 was given 2 points. The history of diabetes was given 1 point. There were three conditions to definition NAFLD: (1) liver steatosis (CAP ≥246 DB/m) [31]; (2) AST >37 U/L or ALT >40 U/L in males and AST or ALT >31 U/L in females [32]; (3) FLI ≥60 [21]. There were three conditions to identified advanced liver fibrosis: (1) VCTE ≥8.8 E/kpa (liver stiffness F3 and F4) [33]; (2) FIB-4 > 3.25[25]; 3) BARD ≥2[34].

### Study variables

The exposure variable in this study was blood selenium. Concentration of blood selenium was measured by Inductively Coupled Plasma Mass Spectrometer (ICP-MS) with Dynamic Reaction Cell Technology (ELAN® DRC II, PerkinElmer Norwalk) using whole blood specimens after a dilution sample preparation step. The lower limit of detection (LLOD) was 24.48 μg/L. All of data were above LLOD.

The covariable in this study involved age, gender, race, education level, smoking status, diabetes, body mass index (BMI), dietary selenium intake and waist circumference. If participants did moderate/vigorous work/recreational activities, they were identified as having physical activity. Smoking status was determined by the participants’ answers of two questions in “Smoking - Cigarette Use” questionnaire – a) Do you now smoke cigarettes? b) Have you smoked at least 100 cigarettes in your entire life? If they didn’t smoke cigarettes now but had ever smoked at least 100 cigarettes, they were defined as former smokers. The following conditions would be defined as diabetes: (a) participants said “Yes”, when doctors asked them “Have you ever been told by a doctor or health professional that you have diabetes or sugar diabetes?” (b) haemoglobin A1C concentration > 6.5%. (c) a fasting plasma glucose level ≥126 mg/dL [35]. The data of dietary selenium intake was obtained from the dietary interview component, called What We Eat in America (WWEIA). The concentration of dietary selenium intake did not include the nutrient from supplements and medications. The details of data can be publicity obtained at http://www.cdc.gov/nchs/nhanes/.

### Statistical analysis

In this study, quantitative variables and qualitative variables were presented as median ± SD and percentage/frequency. Quantitative variables and qualitative variables were analysed by weighted linear regression and weighted chi-square test to calculate differences between four blood selenium groups. Multiple logistic regression model was performed to estimated β and 95% confidential interval associated from blood selenium and NAFLD/advanced liver fibrosis with the first group as reference. Three multivariable models for NAFLD/advanced liver fibrosis were conducted to estimate the clinical interpretation of multiple logistic regression analysis results. Confounding variables which were selected in a series of three additive models included socio-economic factors and recognised risk factors of NAFLD/advanced liver fibrosis. Model 1 was a crude model which was fitted with blood selenium level and NAFLD/advanced liver fibrosis. In model 2, covariables were adjusted for age, gender and race/ethnicity, education level. In model 3, covariables were further adjusted for age, gender, race/ethnicity, BMI, waist circumference, smoking status, physical activity, dietary selenium intake, and diabetes. Subgroup analyses were performed to evaluate the relationship between the blood selenium and NAFLD/advanced liver fibrosis in different subgroups, including age, gender and BMI. The analysis was mainly performed by EmpowerStats software (http://www.empowerstats.com/cn/, X&Y solutions, Inc., Boston, MA). All probability values were two-sided, and a P-value for interaction <0.05 was considered statistically significant.

## Results

### Participants characteristics

In this study, 3340 participants were enrolled in main analysis. They were stratified into four groups according to quartile the concentration of blood selenium (Figure 1). Among these, 835 participants were attributed to Q1 group (>89.8, ≤174.91 μg/L), 835 participants to Q2 group (>174.91, ≤189.005 μg/L), 895 participants to group 3(>189.005, ≤205.32 μg/L), 895 participants to group 4(>205.32, ≤453.62 μg/L). Table 1 shown the baseline characteristics of participants based on four blood selenium strata. Among four groups, gender, race, physical activity, smoking status, dietary selenium intake, ALT and median CAP were all significant difference. Obviously, with higher concentration of blood selenium, there were more male participants and participants had higher dietary selenium intake and activity of serum ALT level and median CAP (Table 1).

**Table 1.** Characteristics of enrolled participants according to blood selenium.

Characteristics	Blood selenium (μg/L)				*P-* Value
	>89.8, ≤174.91	>174.91, ≤189.005	>189.005, ≤205.32	>205.32, ≤453.62	
	(*n* = 833)	(*n* = 835)	(*n* = 833)	(*n* = 835)	
Age, years	51.44 ± 18.24	50.34 ± 17.31	51.17 + 17.06	51.61 ± 16.73	.450
Gender, n (%)					<.001
Male	351 (42.14%)	389 (46.59%)	419 (50.30%)	443 (53.05%)	
Female	482 (57.86%)	446 (53.41%)	414 (49.70%)	392 (46.95%)	
Race, n (%)					<.001
Mexican American	104 (42.14%)	131 (15.69%)	118 (14.17%)	127 (14.19%)	
Other Hispanic	86 (10.32%)	82 (9.82%)	82 (9.84%)	74 (8.86%)	
Non-Hispanic Whit	286 (34.33%)	277 (33.17%)	291 (34.93%)	305 (36.53%)	
Non-Hispanic Black	225 (27.01%)	191 (22.87%)	185 (22.21%)	141 (16.89%)	
Other Race	132 (15.85%)	154 (18.44%)	157 (18.85%)	197 (23.59%)	
Education level, n (%)					.075
≤High school	173 (20.82%)	161 (19.28%)	160 (19.28%)	133 (15.95%)	
>High school	658 (79.18%)	674 (80.72%)	670 (80.72%)	701 (84.05%)	
Physical activity, n (%)					.007
No	288 (34.57%)	246 (29.46%)	232 (27.85%)	232 (27.78%)	
Yes	545 (65.43%)	589 (70.54%)	601 (72.15%)	603 (72.22%)	
Smoking status, n (%)					.015
Never	459 (55.10%)	504 (60.36%)	517 (62.06%)	514 (61.56%)	
Former	243 (29.17%)	222 (26.59%)	220 (26.41%)	233 (27.90%)	
Current	131 (15.73%)	109 (13.05%)	96 (11.52%)	88 (10.54%)	
Diabetes, n (%)					.229
No	721 (80.65%)	717 (80.02%)	695 (77.65%)	693 (77.43%)	
Yes	173 (19.35%)	179 (19.98%)	200 (22.35%)	202 (22.57%)	
BMI(kg/m^2^)	29.55 ± 7.43	29.84 ± 7.10	29.84 ± 6.84	29.72 ± 6.67	.821
Waist circumference (cm)	99.96 ± 17.54	100.41 ± 16.70	101.00 ± 16.18	101.09 ± 16.30	.464
Dietary selenium intake (ug)	105.98 ± 51.72	108.81 ± 53.39	109.67 ± 53.04	113.81 ± 57.35	.029
ALT (IU/L)	19.24 ± 12.93	21.62 ± 14.84	22.58 ± 14.43	24.41 ± 17.78	<.001
AST (IU/L)	20.83 ± 10.59	21.20 ± 10.47	21.23 ± 8.84	21.64 ± 9.85	.431
Median stiffness E/kpa	5.99 ± 5.87	5.70 ± 4.58	5.72 ± 4.36	5.55 ± 2.43	.237
Median CAP DB/m	258.02 ± 61.86	261.48 ± 61.39	269.89 ± 61.35	274.62 ± 63.42	<.001

BMI: Body Mass Index; ALT: Alanine aminotransaminase; AST: Aspartate aminotransferase; CAP: Controlled Attenuation Parameter.

### Participants with NAFLD presented a significantly higher blood selenium level

Multiple logistic regression models were constructed to explore whether the concentration of blood selenium was independent associated with NAFLD diagnosed by VCTE (Table 2). In model 1, no variables were adjusted. Compared to Q1 group, high blood selenium level (Q3 and Q4 group) had significantly positive association with NAFLD (β = 1.34, 95%Cl, 1.10–1.63 and 1.45, 95%Cl, 1.19–1.77, *P* for trend <.01). In model 2, age, gender and race/ethnicity, education level were further adjusted. Compared to referent group, high blood selenium (Q3 and Q4 group) remained significantly positive association with NAFLD (β = 1.30, 95% CI, 1.06–1.59 and 1.36 95% CI, 1.11–1.67, *P* for trend <.01). To further exclude the influence of covariables, age, gender, race/ethnicity, BMI, waist circumference, smoking status, physical activity, dietary selenium intake, and diabetes were adjusted in model 3. The highest blood selenium level still had significant positive association with NAFLD (β = 1.31, 95% CI, 1.03–1.67, *P* for trend <.01). In addition, subgroups analysis of age, gender and BMI were conducted to identify the independent relationship between blood selenium level and NAFLD diagnosed by VCTE. But there was no significant distinction relationship of blood selenium level and NAFLD between subgroups divided by age, gender, BMI and dietary selenium intake (Table 3). To further prove the correlation between blood selenium level and NAFLD, NAFLD was re-defined by serum activity of AST and ALT and FLI (Supplementary Table 1). However, high blood selenium level was just significant positive associated with NAFLD diagnosed by serum activity of AST and ALT in model 1(β = 1.42, 95% CI, 1.05–1.93, and 1.45, 95% CI, 1.07–1.97, *P* for trend = .01). Furthermore, compared to diagnosed by VCTE, high blood selenium level was equally significant association to NAFLD diagnosed by FLI in model 1 (β = 1.38, 95% CI, 1.06–1.80 and 1.45, 95% CI, 1.11–1.88, *P* for trend <.01), model 2 (β = 1.38, 95% CI, 1.06–1.81 and 1.48, 95% CI, 1.13–1.94, *P* for trend <.01), model 3 (β = 1.65, 95% CI, 1.01–2.72, *P* for trend = .01)

**Table 2.** Association between blood selenium level and NAFLD diagnosed by vibration controlled transient elastography.

	Model 1	Model 2	Model 3
	β (95% CI)	β (95% CI)	β (95% CI)
Blood selenium level (ug/L, quartile)			
Q1	Reference	Reference	Reference
Q2	1.03 (0.85, 1,25)	1.00 (0.82, 1.22)	0.94 (0.74, 1.19)
Q3	1.34 (1.10, 1.63)	1.30 (1.06, 1.59)	1.23 (0.97, 1.57)
Q4	1.45 (1.19, 1.77)	1.36 (1.11, 1.67)	1.31 (1.03, 1.67)
P for trend	<0.01	<0.01	<0.01

Model 1: no covariates were adjusted. Model 2: age, gender and race/ethnicity, education level were adjusted. Model 3: age, gender, race/ethnicity, BMI, waist circumference, smoking status, physical activity, dietary selenium intake and diabetes were adjusted.

**Table 3.** Subgroup analyses of the association between blood selenium level and NAFLD diagnosed by vibration controlled transient elastography.

		Blood selenium (μg/L)			
		Q1	Q2	Q3	Q4
	n	β(95% CI) *P*-value	β(95% CI) *P*-value	β(95% CI) *P*-value	β(95% CI) *P*-value
Age, years					
<53	1643	Reference	1.01 (0.77, 1.33) .924	1.45 (1.10, 1.91) .008	1.44 (1.09, 1.90) .009
≥53	1693	Reference	1.07 (0.80, 1.42) .640	1.20 (0.91, 1.60) .197	1.45 (1.09, 1.93) .012
Gender					
Male	1602	Reference	0.82 (0.61, 1.09) .175	1.22 (0.91, 1.64) .181	1.52 (1.13, 2.05) .005
Female	1734	Reference	1.21 (0.94, 1.57) .147	1.37 (1.05, 1.79) .019	1.27 (0.97, 1.67) .077
BMI					
≥29.9	1422	Reference	1.00 (0.70, 1.44) .987	1.20 (0.83, 1.74) .228	1.38 (0.94, 2.03) .102
≥24.9, <29.9	1069	Reference	0.98 (0.69, 1.40) .932	1.35 (0.95, 1.93) .096	1.53 (1.08, 2.17) .018
<24.9	840	Reference	0.99 (0.63, 1.55) .968	1.38 (0.89, 2.15) .155	1.45 (0.93, 2.26) .101
Dietary Selenium Intake (ug/d)					
≥2.2, <99.25	1668	Reference	1.08 (0.83, 1.41) .567	1.22 (0.93, 1.61) .008	1.40 (1.06, 1.90) .009
≥99.3, <467.2	1668	Reference	0.97 (0.74, 1.29) .853	1.46 (1.10, 1.94) .197	1.48 (1.12, 1.96) .012

BMI: Body Mass Index.

### Participant with lower blood selenium level showed higher percentage of advanced liver fibrosis

Advanced liver fibrosis was the important pathological manifestation of late stage NAFLD and had seriously influence of NAFLD patients’ prognosis. So, multiple logistic regression models also were conducted to explore whether blood selenium level was independent associated advanced liver fibrosis diagnosed by VCTE (Table 4). In model 1, compared referent group, high blood selenium level (Q3 group) was significantly inversely association with advanced liver fibrosis (β = 95% CI 0.46–0.93, *P* for trend = .04). In model 2, high blood selenium (Q3 and Q4 group) remained significantly inversely association with advanced liver fibrosis with age, gender and race were adjust. (β = 0.64, 95% CI 0.44–0.92 and 0.67 95% CI, 0.47–0.96, *P* for trend = .03) To further exclude the influence of covariables, age, gender, race/ethnicity, BMI, waist circumference, smoking status, physical activity, dietary selenium intake and diabetes were adjusted in model 3. The high blood selenium level (Q2, Q3 and Q4 group) still had significant inversely association with advanced liver fibrosis (β = 0.65, 95% CI, 0.44–0.96; 0.59, 95% CI 0.40–0.88 and 0.61 95% CI, 0.41–0.90, *P* for trend = .01). Furthermore, subgroups analysis of age, gender, BMI and dietary selenium intake were conducted to identify the relationship between blood selenium level and advanced liver fibrosis (Table 5). Interestingly, compared to women, men contributed the main inversely correlation between blood selenium and advanced liver fibrosis in Q2 group (0.41, 95% CI, 0.25–0.68 vs 1.20, 95% CI, 0.72–2.01), Q3 group (0.43, 95% CI, 0.27–0.70 vs 1.01, 95% CI, 0.59–1.74) and Q4 group (0.47, 95% CI, 0.29–0.75 vs 1.07, 95% CI, 0.62–1.84). For further conforming the negative association between blood selenium level and advanced liver fibrosis, we used another two indexes index to diagnose advanced liver fibrosis. Diagnosed by FIB-4, high blood selenium was also the negative association with advanced liver fibrosis in model 1 (compare to Q1 group, Q2 group: β = 0.45, 95% Cl, 0.21–0.95, Q4 group: β = 0.31, 95% Cl, 0.13–0.74, *P* for trend <.01), model 2 (compare to Q1 group, Q4 group: β = 0.35, 95% Cl, 0.15–0.84, *P* for trend = .02), model 3 (compare to Q1 group, Q4 group: β = 0.32, 95% Cl, 0.13–0.77, *P* for trend = .01). Similar to those diagnosed by VCTE and FIB-4, high blood selenium level was equally negative association with advanced fibrosis liver in model 1 (β = 0.78, 95% Cl, 0.65–0.94, 0.71, 95% Cl, 0.59–0.85 and 0.63, 95% Cl, 0.53–0.76, *P* for trend <.01), model 2 (compare to Q1 group, Q3 group: β = 0.78, 95% Cl, 0.64–0.94, Q4 group: β = 0.73, 95% Cl, 0.60–0.88, *P* for trend <.01), model 3 (β = 0.71, 95% Cl, 0.57–0.89, 0.62, 95% Cl, 0.49–0.77 and 0.59, 95% Cl, 0.47–0.73, *P* for trend <.01).

**Table 4.** Association between blood selenium level and advanced liver fibrosis diagnosed by vibration controlled transient elastography.

	Model 1	Model 2	Model 3
	β (95% CI)	β (95% CI)	β (95% CI)
Blood selenium level (ug/L, quartile)			
Q1	Reference	Reference	Reference
Q2	0.70 (0.49, 1.00)	0.70 (0.49, 1.00)	0.65 (0.44, 0.96)
Q3	0.65 (0.46, 0.93)	0.64 (0.44, 0.92)	0.59 (0.40, 0.88)
Q4	0.70 (0.49, 1.00)	0.67 (0.47, 0.96)	0.61 (0.41, 0.90)
P for trend	0.04	0.03	0.01

Model 1: no covariates were adjusted. Model 2: age, gender and race/ethnicity, education level were adjusted. Model 3: age, gender, race/ethnicity, BMI, waist circumference, smoking status, physical activity, dietary selenium intake and diabetes were adjusted.

**Table 5.** Subgroup analyses of the association between blood selenium level and advanced fibrosis diagnosed by vibration controlled transient elastograph.

		Blood selenium (μg/L)			
		Q1	Q2	Q3	Q4
	n	β(95% CI) *P-*value	β(95% CI) *P*-value	β(95% CI) *P*-value	β(95% CI) *P*-value
Age, years					
<53	1643	Reference	0.88 (0.48, 1.62) .685	0.92 (0.49, 1.70) .780	1.00 (0.55, 1.82) .999
≥53	1693	Reference	0.63 (0.41, 0.98) .040	0.53 (0.34, 0.83) .005	0.57 (0.36, 0.88) .012
Gender					
Male	1602	Reference	0.41 (0.25, 0.68) <.001	0.43 (0.27, 0.70) <.001	0.47 (0.29, 0.75) .002
Female	1734	Reference	1.20 (0.72, 2.01) .478	1.01 (0.59, 1.74) .972	1.07 (0.62, 1.84) .806
BMI					
≥29.9	1422	Reference	0.81 (0.53, 1.23) .318	0.80 (0.53, 1.21) .298	0.67 (0.43, 1.04) .074
≥24.9, <29.9	1069	Reference	0.58 (0.24, 1.39) .225	(0.11, 0.88) .028	0.75 (0.34, 1.65) .475
<24.9	840	Reference	0.22 (0.05, 1.01) .051	0.12 (0.02, 0.95) .045	0.77 (0.28, 2.16) .623
Dietary Selenium Intake (ug/d)					
≥2.2, <99.25	1668	Reference	0.88 (0.48, 1.62) .685	0.92 (0.49, 1.70) .780	1.00 (0.55, 1.82) .999
≥99.3, <467.2	1668	Reference	0.63 (0.41, 0.98) .040	0.53 (0.34, 0.83) .005	0.57 (0.36, 0.88) .012

BMI: Body Mass Index.

## Discussion

We conducted this study to explore the significant association between blood selenium and NAFLD/advanced liver fibrosis. Thereinto, NAFLD was considered as one of metabolic diseases [36]. Metabolic syndrome was its strongest risk syndrome, which characterised as increased BMI, waist circumference, impaired fasting glucose and diabetes [37]. Moreover, recent studies estimated current smoking and sedentary behaviour/low physical activity were independent risk factors of NAFLD [38,39]. In the obese rat, smoking would promote the progress of NAFLD *via* hypercholesterolaemia, insulin resistance, and liver lipogenesis [40]. In addition, physical activity can attenuate ROS-induced oxidative stress in NAFLD [41].

Selenium showed a pivotal role in numerous diseases, especially metabolic diseases [42]. But The relationship between selenium and NAFLD still needs further clarification. Usually, Selenium was recognized as antioxidants to attenuate the development of NAFLD [43]. Yang Yi [44] and Seyedeh [45] *et al.* found high selenium exposure decreased liver steatosis, HOMA-IR, LDL/HDL-c and TC/HDL-c ratios which were aetiologies of NAFLD in NAFLD rodent models *via* directly suppling high-fat diets (HBD) with added selenomethionine. Moreover, the increase of blood selenium and selenoproteins in NAFLD mice model attenuated inflammation, lipogenesis, dysfunction of lipid metabolism, and oxidative stress, retarded progression of simple steatosis to NASH, even liver fibrosis and cirrhosis [46]. However, several clinical studies suggested that high selenium exposure had a positive relationship of NAFLD [16,17,47]. So, we further evaluated association between blood selenium level and NAFLD diagnosed by VCTE in American.

Similarly, our study indicated that high level of blood selenium was associated with NAFLD. We suggested that high blood selenium level may lead hyperglycaemia, hyperinsulinemia and hyperlipidaemia with increasing expression of lipogenesis associated proteins [48,49]. In addition, pigs with elevation blood level of selenium showed downregulation of INSR (insulin receptor) and upregulation of mTOR-s6, regarded as insulin resistance [49]. In GPxs (one typical selenoprotein) overexpressing mice, GPxs excessively diminished reactive oxidative species including H_2_O_2_ and lead to decrease phosphorylation of insulin receptors β-subunit and Akt, which was the main cause of desensitive insulin signalling and active lipogenesis probably [50]. Andreas [51] *et al.* thought high selenium exposure would raise maximum expression and activity of GPxs compared to lack of selenium and enhance activity of PTP1b which further upgrade SREBP-1c that the key molecular of liver lipogenesis and accumulation. In spite of that, the effect and mechanism of elevated blood selenium on NAFLD are still doubtful, and more rigorous animal experiments and clinical experiments are needed to explore.

Several experiments indicated selenium can extenuate liver fibrosis. Responded to fibrotic stimulations in NAFLD, hepatic stellate cells (HSCs), the main source of hepatic fibrogenesis, elevated proliferation, survival, viability, migration, synthesis and remodelling extracellular matrix (ECM) [52]. Low selenium level drove upregulation of MMP1/3 and periportal fibrosis in rat’s liver [53]. Reversely, high selenium level elevated apoptosis level of HSCs, upregulated expression of TIMP-1 and alleviated cellular viability of HSCs, downregulation of collagens, TGF-β, hydroxyproline *via* downregulation of silent information regulator (SIRT)1 and mitogen-activated protein kinase (MAPK) signalling in CCl4 induced liver injury and fibrosis [54–56]. As well, Luan [57] *et al.* reported that hydrogen selenide led to degradation of collagen IV *via* uncoupling the sulfilimine bond to reduce remodelling of ECM, regarded as attenuating liver fibrosis. In liver fibrosis mice model, high selenium level elevated expression and activity of GPx and superoxide dismutase with decreasing level of MDA, TNF-α, IL-6 and MCP-1 [55]. Therefore, eliminating inflammation and oxidative stress maybe the potential mechanism by which high selenium level reduces liver fibrosis.

Nonetheless, the existing clinical trial enrolled participants in last contrary and diagnosed liver fibrosis by indirect methods. With the changes of nutrition situation in several two decades, these results can’t the real situation in current people. So, we observed participants in NHAENS 2017-2018 and diagnosed liver fibrosis by VCTE. In our study, participants with lower blood selenium level showed higher percentages of advanced liver fibrosis, especially among males. Therefore, we suggested that high blood selenium could be a significant protective factor for advanced fibrosis in NAFLD patients.

It has been clarified that dietary selenium intake can regulate the progress of NAFLD, even liver fibrosis in animal experiments and clinical trials [15]. So, we adjusted the effect of dietary selenium intake and conducted subgroup analysis. In spite of that, high blood selenium still showed the positive association to NAFLD and negative association to advanced liver fibrosis. On the one hand, dietary selenium intake of U.S. adults far exceeds that required to achieve the maximum plasma selenoprotein level [9]. Thus, dietary selenium intake has little effect on blood selenium level in U.S. adults. On the other hand, the results suggested that the effect of elevated blood selenium on NAFLD and advanced liver fibrosis was caused by the imbalance of selenium homeostasis in the body rather than dietary selenium intake.

This study explored the association between blood selenium level and NAFLD through a large sample cross-sectional research and adjusted for potential cofounding variables to enhance validity of the results. While it still remains some limitations. Firstly, this study was an observational study which just investigated the relationship of high blood selenium level and NAFLD/liver fibrosis. It needed more prospective studies to clarify the exposure risk of blood selenium in NAFLD and advanced liver fibrosis. Secondly, although we strived to adjust every covariable, there still remain variables that affected the association between blood selenium and NAFLD/liver fibrosis. Thirdly, there was no consensus on the cut-off for diagnosis of liver steatosis and fibrosis by VCTE and activity of ALT & AST, diabetes, excessive alcohol consumption. We would conduct a cohort study to further validate our findings.

## Conclusions

In summary, this study showed that blood selenium level had a significant positive association to NAFLD diagnosed by VCTE among US population. Participants with lower blood selenium level showed higher percentage of advanced liver fibrosis. Blood selenium is more likely to cause NAFLD and liver fibrosis due to imbalances in selenium homeostasis rather than dietary selenium intake. It provides a new perspective for the pathogenesis of NAFLD and advanced fibrosis.

## Supplementary Material

Supplemental Material

## Data Availability

The data used in this study are from a public database at https://www.cdc.gov/nchs/nhanes/index.htm, which can be accessed by everyone through the links provided in the paper.
