# Supplementary material for: The association between non-alcoholic fatty liver disease (NAFLD) and advanced fibrosis with blood selenium level based on the NHANES 2017-2018
Source: Ann Med. 2022 Aug 17;54(1):2259–68. doi: 10.1080/07853890.2022.2110277 (PMC9455329; doi:10.1080/07853890.2022.2110277)
Supplement: Supplemental Material [file IANN_A_2110277_SM8767.pdf]

Appendices

Supplementary table 1: Association between blood selenium level and NAFLD diagnosed by ALT&AST and FLI.

	NAFLD diagnosed by AST&ALT			NAFLD diagnosed by FLI		
	Model 1<br>β (95 CI)	Model 2<br>β (95 CI)	Model 3<br>β (95 CI)	Model 1<br>β (95 CI)	Model 2<br>β (95 CI)	Model 3<br>β (95 CI)
Blood selenium level (ug/L, quartile)						
Q1	Reference	Reference	Reference	Reference	Reference	Reference
Q2	1.23 (0.89, 1.68)	1.16 (0.85, 1.60)	1.15 (0.83, 1.59)	1.07 (0.83, 1.40)	1.09 (0.83, 1.42)	0.88 (1.52, 1.47)
Q3	1.42 (1.05, 1.93)	1.36 (0.99, 1.85)	1.33 (0.97, 1.82)	1.38 (1.06, 1.80)	1.38 (1.06, 1.81)	1.65 (1.01, 2.72)
Q4	1.45 (1.07, 1.97)	1.35 (0.99, 1.85)	1.30 (0.95, 1.79)	1.45 (1.11, 1.88)	1.48 (1.13, 1.94)	1.65 (1.00, 2.72)
P for trend	0.01	0.04	0.09	<0.01	<0.01	0.01

Model 1: no covariates were adjusted. Model 2: age, gender and race/ethnicity, education level were adjusted. Model 3: age, gender, race/ethnicity, BMI, waist circumference, smoking status, physical activity, dietary selenium intake and diabetes were adjusted. NAFLD: Non-alcoholic fatty liver disease; ALT: Alanine aminotransferase; AST: Aspartate aminotransferase; FLI: Fatty liver index

For Peer Review Only

Supplementary table 2: Association between blood selenium level and advanced liver fibrosis diagnosed by FIB-4 and BARD.

	FIB-4			BARD		
	Model 1<br>β (95 CI)	Model 2<br>β (95 CI)	Model 3<br>β (95 CI)	Model 1<br>β (95 CI)	Model 2<br>β (95 CI)	Model 3<br>β (95 CI)
Blood selenium level (ug/L, quartile)						
Q1	Reference	Reference	Reference	Reference	Reference	Reference
Q2	0.45 (0.21, 0.95)	0.51 (0.24, 1.11)	0.46 (0.21, 1.00)	0.78 (0.65, 0.94)	0.83 (0.69, 1.00)	0.71 (0.57, 0.89)
Q3	0.54 (0.27, 1.10)	0.62 (0.30, 1.30)	0.59 (0.28, 1.24)	0.71 (0.59, 0.85)	0.78 (0.64, 0.94)	0.62 (0.49, 0.77)
Q4	0.31 (0.13, 0.74)	0.35 (0.15, 0.84)	0.32 (0.13, 0.77)	0.63 (0.53, 0.76)	0.73 (0.60, 0.88)	0.59 (0.47, 0.73)
P for trend	<0.01	0.02	0.01	<0.01	<0.01	<0.01

Model 1: no covariates were adjusted. Model 2: age, gender and race/ethnicity, education level were adjusted. Model 3: age, gender, race/ethnicity, BMI, waist circumference, smoking status, physical activity, dietary selenium intake and diabetes were adjusted. FIB-4: Fibrosis-4 index; BARD: Body mass index, AST/ALT Ratio, Diabetes score

For Peer Review Only
